# Proximal Protection Technique for Migrated Closed-Cell Stents in Carotid Artery Stenting

**DOI:** 10.7759/cureus.78552

**Published:** 2025-02-05

**Authors:** Satoshi Anai, Atsushi Ogata, Takashi Furukawa, Tatsuya Abe

**Affiliations:** 1 Neurosurgery, Faculty of Medicine, Saga University, Saga, JPN

**Keywords:** carotid artery stenting (cas), microballoon, proximal protection, stent migration, vascular intervention

## Abstract

This case report details an innovative management strategy for a migrated and shortened closed-cell stent in carotid artery stenting (CAS). A 78-year-old male underwent CAS, which was initially successful. However, stent migration the following day complicated the outcome, obstructing the external carotid artery (ECA). We addressed this by navigating a Scepter XC microballoon (MicroVention, Tustin, CA) through the stent's cells to occlude the ECA and utilizing a balloon-guiding catheter in the common carotid artery for proximal protection. This approach enabled successful additional CAS without complications. Our technique highlights the importance of adaptable strategies in managing rare CAS complications.

## Introduction

Carotid artery stenting (CAS) has emerged as an alternative to carotid endarterectomy (CEA) for treating carotid stenosis. While CAS is less invasive and associated with fewer systemic complications, it carries a higher risk of perioperative ischemic stroke compared to CEA [1,2]. Embolic protection devices (EPDs) play a crucial role in reducing the risk of stroke during CAS procedures [3,4]. Recent studies suggest that CAS with proximal protection devices can be effective and safe for treating unstable plaques. Proximal protection involves blocking blood flow in the external and common carotid arteries, reducing the risk of embolic complications [5,6].

Postoperative stent migration is a rare complication of CAS that can require the placement of additional stents [7-9]. While there are no reported methods of proximal protection that involve occluding both the external and common carotid arteries when a migrated stent compromises the external carotid artery (ECA), this report introduces a novel approach. We describe a case where a microballoon was navigated through the struts of a migrated stent into the ECA, providing effective proximal protection during CAS.

## Case presentation

**Figure 1:**
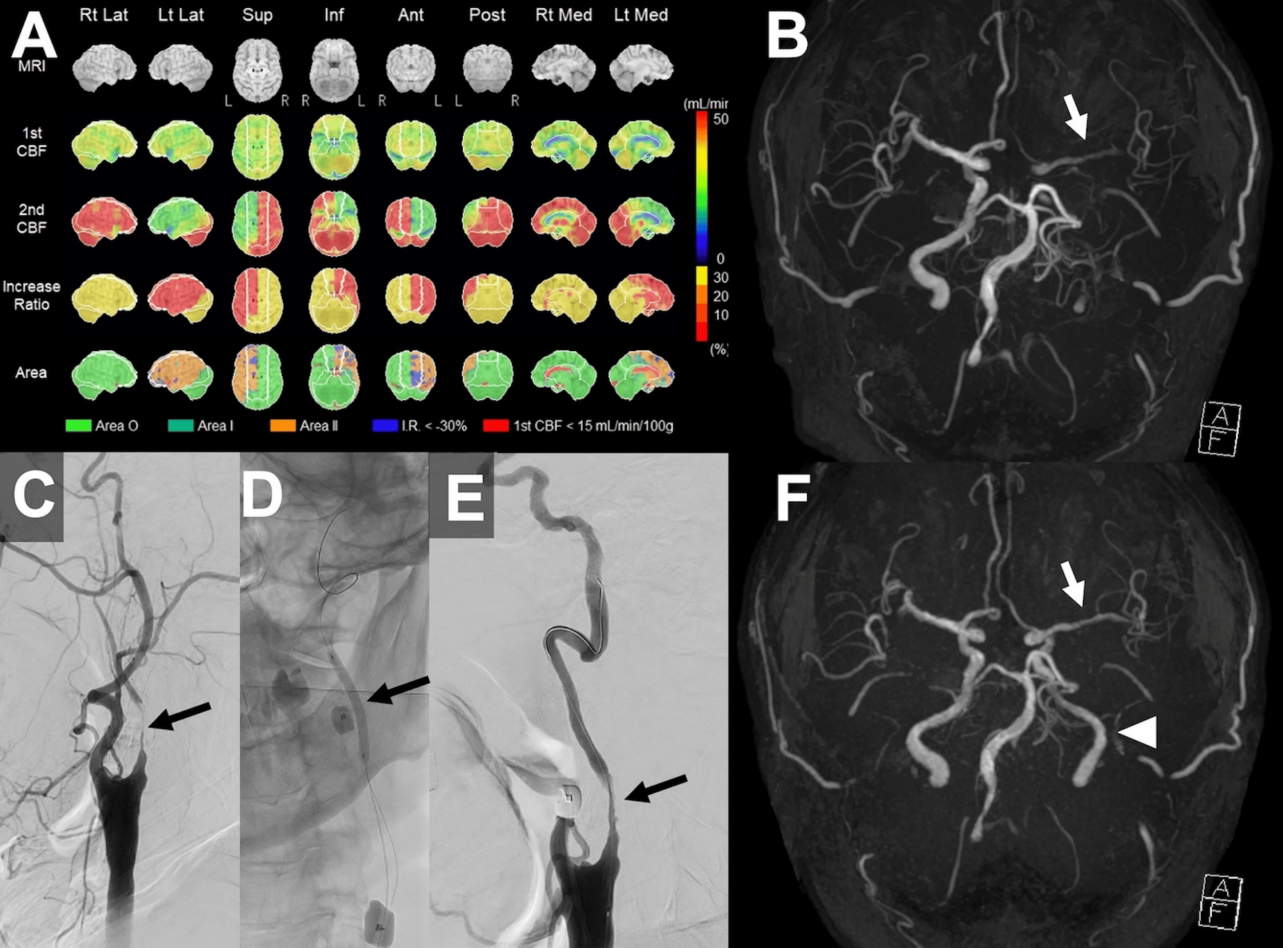
Preoperative perfusion imaging, initial angioplasty, and sequential magnetic resonance angiography. (A) Iodine-123 N-isopropyl-p-iodoamphetamine single-photon emission computed tomography (IMP-SPECT) demonstrating decreased cerebral perfusion and impaired vascular reserve in the left hemisphere. (B) Time-of-flight magnetic resonance angiography (TOF-MRA) showing attenuated signal intensity in the left middle cerebral artery compared to the contralateral side. (C) Digital subtraction angiography of the left carotid artery revealing high-grade stenosis of the internal carotid artery. (D) Endovascular intervention with percutaneous transluminal angioplasty (PTA) performed under proximal protection. (E) Post-interventional angiogram showing modest luminal gain at the site of stenosis. (F) Post-interventional TOF-MRA depicting improved flow signal in the left internal carotid artery (arrowhead) and middle cerebral artery (arrow).

**Figure 2:**
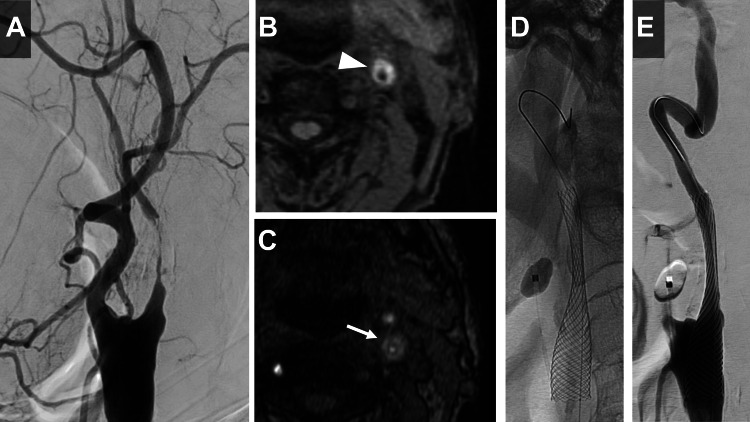
Preoperative plaque characterization and initial carotid artery stenting procedure. (A) Left carotid angiogram showing severe stenosis in the internal carotid artery. (B) Magnetization-prepared rapid acquisition with gradient echo (MP-RAGE) image showing a hyper-intense plaque (white arrowhead). (C) Magnetic resonance angiography-time of flight (MRA-TOF) imaging also reveals a hyper-intense plaque (white arrow). (D) Carotid artery stenting (CAS) is performed with a Carotid Wallstent under proximal protection. (E) Post-procedural left carotid angiogram demonstrating successful expansion of the stenosed lesion.

A 78-year-old male developed right hemiparesis and was found to have symptomatic left internal carotid artery (ICA) stenosis. He was referred to our hospital for CAS. Upon admission, his right hemiparesis had resolved, and no significant neurological abnormalities were noted. Cerebral angiography revealed high-grade stenosis of 95% at the origin of the left ICA per the North American Symptomatic Carotid Endarterectomy Trial (NASCET) criteria (Figure 2A), and MRI plaque imaging showed an unstable plaque with high signal intensity on both magnetization-prepared rapid acquisition with gradient echo (MP-RAGE) and magnetic resonance angiography (MRA)-time of flight (TOF) sequences (Figures 2B, 2C). Digital subtraction angiography revealed isolated leptomeningeal collateralization from the left posterior cerebral artery territory to the anterior and middle cerebral artery territories. Iodine-123 N-isopropyl-p-iodoamphetamine single-photon emission computed tomography (IMP-SPECT) performed for cerebral blood flow assessment diagnosed a high risk of hyperperfusion syndrome due to reduced resting blood flow and vascular reactivity in the left cerebral hemisphere (Figure 1A). Preoperative MRA-TOF demonstrated markedly decreased signal intensity in the left middle cerebral artery (MCA) compared to the contralateral side (Figure 1B). To prevent hyperperfusion syndrome, staged angioplasty was planned. The unstable nature of the plaque raised concerns about cerebral embolism during percutaneous transluminal angioplasty (PTA) and CAS, prompting the decision to employ proximal protection.

The initial treatment involved PTA under local anesthesia. A 9Fr sheath was placed in the right femoral artery, and a 3 mm diameter balloon was used for PTA under proximal protection with a 9Fr Mo.Ma Ultra Proximal Cerebral Protection Device (Medtronic, Minneapolis, MN) (Figures 1C-1E). Following PTA, MRA revealed substantial improvement in signal intensity of the left ICA and MCA compared to preoperative imaging (Figure 1F), indicating restoration of cerebral perfusion in the left hemisphere. No symptomatic complications were observed postoperatively.

**Figure 3:**
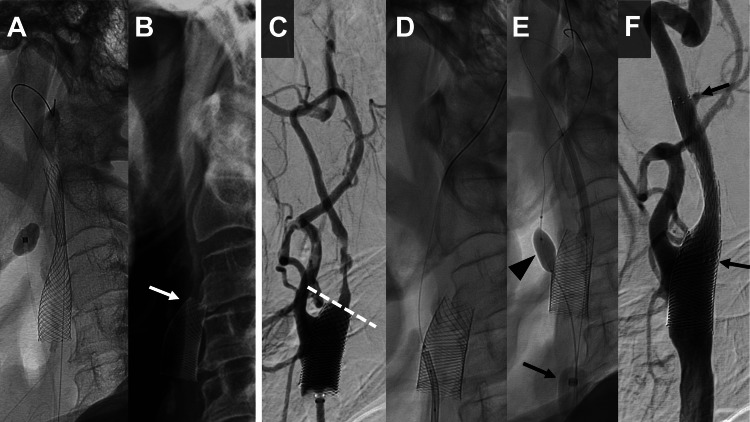
Stent migration and subsequent endovascular intervention with proximal protection technique. (A) X-ray immediately after carotid artery stenting (CAS) showing the Carotid Wallstent in the correct position. (B) X-ray taken one day after CAS illustrating the shortened Carotid Wallstent and its proximal migration (white arrow). (C) Pre-additional stenting left carotid angiogram showing the migrated stent covering the origin of the external carotid artery (ECA), with the distal end outlined by a dotted white line. (D) Micro guidewire navigated through the strut of the Carotid Wallstent. (E) Inflation of the Scepter XC microballoon through the cells of the Carotid Wallstent into the ECA (black arrowhead) and simultaneous inflation of the balloon-guiding catheter in the common carotid artery (CCA) (black arrow). This setup enabled occlusion of both the ECA and CCA, allowing CAS under proximal protection. (F) Left carotid angiogram post additional CAS displaying successful expansion of the previously stenosed region. Black arrows indicate added stent.

One month later, CAS was performed. MP-RAGE imaging demonstrated plaque instability with a plaque-to-muscle signal intensity ratio of 2.03, which led to the selection of a closed-cell stent design for CAS. Pre-dilatation was done with a 3.5 × 30 mm PTA balloon SHIDEN (Kaneka, Osaka, Japan) under proximal protection by Mo.Ma Ultra Proximal Cerebral Protection Device, followed by the placement of a 10 × 31 mm Carotid Wallstent (Boston Scientific, Marlborough, MA) (Figure 2D), achieving good expansion (Figure 2E). Given the common carotid artery diameter of 10.6 mm, incomplete stent apposition was observed at its proximal segment. No symptomatic complications were observed postoperatively. However, the next day (postoperative day one), plain X-rays revealed shortening and proximal migration of the stent (Figures 3A, 3B), necessitating a repeat CAS. There were no associated cerebral infarcts. The left carotid angiogram showed a shortened stent covering the origin of the ECA (Figure 3C). Since the same Mo.Ma Ultra Proximal Cerebral Protection Device could not be used as in previous treatments, a balloon-guiding catheter (Branchor, Asahi Intecc, Aichi, Japan) was employed to occlude the common carotid artery, and a microballoon, Scepter XC (4 × 11 mm) (MicroVention, Tustin, CA), was used to occlude the ECA under proximal protection. A 9F balloon-guiding catheter (Branchor) and a 5F guiding catheter (ENVOY MPD, Codman, Raynham, MA) were inserted into the common carotid artery. A Scepter XC microballoon and a micro guidewire (CHIKAI, Asahi Intecc, Aichi, Japan) were introduced through the 5F guiding catheter and navigated through the stent struts (Figure 3D). This setup allowed for the occlusion of both the external and common carotid arteries, and PTA was performed under proximal protection (Figure 3E). The 5-French guiding catheter was withdrawn proximal to the balloon in the common carotid artery to avoid catheter-balloon interference during proximal protection. Subsequently, a PROTAGE tapered stent (10-7 × 40 mm) (Medtronic, Minneapolis, MN) was overlaid on the shortened stent, achieving good expansion (Figure 3F). The patient was discharged without any symptomatic complications on the sixth postoperative day.

## Discussion

In this case, a closed-cell Carotid Wallstent exhibited shortening and migration the day after CAS. The exact cause and mechanism of stent migration are unknown, but the possibility of a watermelon-seeding effect, which is exacerbated by a significant gradient between proximal and distal vessel diameters, has been identified as a contributing anatomical factor [8]. In this case, there was a notable difference in the diameters of the internal and common carotid arteries, which aligns with this theory. In retrospect, to prevent stent migration, the longer stents or multiple overlapping stents could have provided better anchoring and stability, especially given the anatomical characteristics that were predisposed to the "watermelon-seeding" effect [8]. Migration can occur immediately post procedure or several months later [8]. While migration is more commonly reported with closed-cell stents, it can also occur with open-cell designs [9,10]. To mitigate these complications, long-term follow-up with imaging is recommended.

Due to the migrated stent covering the origin of the ECA, the insertion of the distal balloon of the Mo.Ma Ultra Proximal Cerebral Protection Device into the ECA was deemed to carry a high risk of stent fracture. Given the presence of an unstable plaque and the associated risk of cerebral embolism, additional CAS was performed using proximal protection. We decided to navigate the Scepter XC microballoon through the cells of the Carotid Wallstent into the ECA to occlude it. Additionally, the common carotid artery (CCA) was occluded using a balloon guide catheter (BGC) guided along a different axis to establish proximal protection. The passability of the Scepter XC through the cells of the Carotid Wallstent was evaluated; the free cell area of the Carotid Wallstent is 1.08 mm² [11,12], and the outer diameter of the Scepter XC tip is 2.6Fr (approximately 0.87 mm), which translates to an area of about 0.6 mm². Thus, it was feasible for the Scepter XC to pass through the free cell area of the Carotid Wallstent. According to Sakamoto et al., the geometry and dimensions of the Carotid Wallstent's free cell area vary with the deployed vessel diameter, with the minor axis of the cell ranging from 1.3 to 2.7 mm [13]. These dimensional characteristics also suggest the feasibility of crossing the Carotid Wallstent's cells with the Scepter XC balloon catheter. Furthermore, the insertion angle into the stent has been reported to significantly affect the success rate [14]. We selected the ENVOY MPD as the guiding catheter for the Scepter XC, ensuring that the micro guidewire and Scepter XC could enter as perpendicular to the stent surface as possible, which was a critical factor in the successful trans-cell navigation.

In the present case, the use of the Scepter XC enabled occlusion of the ECA and the use of a BGC for the CCA provided proximal protection, allowing for PTA and stent placement without causing distal embolization. However, if the Scepter had not been able to pass through the Carotid Wallstent, a more invasive alternative involving retrograde guidance of a catheter from the superficial temporal artery (STA) to the ECA would have been considered as an option [15,16].

## Conclusions

This case demonstrates the importance of careful stent selection and deployment strategy in carotid artery stenting, particularly when encountering significant vessel diameter mismatch that may predispose to stent migration. When stent migration occurs, the technique of navigating a microballoon through stent cells for external carotid artery occlusion, combined with proximal protection, can provide a viable solution for additional stenting procedures.
